# Inhibition of CD40-TRAF6 interactions by the small molecule inhibitor 6877002 reduces neuroinflammation

**DOI:** 10.1186/s12974-017-0875-9

**Published:** 2017-05-12

**Authors:** Suzanne A. B. M. Aarts, Tom T. P. Seijkens, Pascal J. H. Kusters, Susanne M. A. van der Pol, Barbara Zarzycka, Priscilla D. A. M. Heijnen, Linda Beckers, Myrthe den Toom, Marion J. J. Gijbels, Louis Boon, Christian Weber, Helga E. de Vries, Gerry A. F. Nicolaes, Christine D. Dijkstra, Gijs Kooij, Esther Lutgens

**Affiliations:** 1 Department of Medical Biochemistry, Subdivision of Experimental Vascular Biology, Academic Medical Center, University of Amsterdam, Meibergdreef 15, 1105 AZ Amsterdam, The Netherlands; 2 Department of Molecular Cell Biology and Immunology, VU University Medical Center, 1007 MB Amsterdam, The Netherlands; 3 Department of Biochemistry, University of Maastricht, 6200 MD Maastricht, The Netherlands; 4 Department of Pathology and Department of Molecular Genetics, Cardiovascular Research Institute Maastricht (CARIM), University of Maastricht, Maastricht, The Netherlands; 5 Bioceros, 3584 CM Utrecht, The Netherlands; 6 Institute for Cardiovascular Prevention (IPEK), Ludwig Maximilians University (LMU), Pettenkoferstraße 9, 80336 Munich, Germany

**Keywords:** Multiple sclerosis, EAE, Co-stimulation, Monocytes, Inflammation

## Abstract

**Background:**

The influx of leukocytes into the central nervous system (CNS) is a key hallmark of the chronic neuro-inflammatory disease multiple sclerosis (MS). Strategies that aim to inhibit leukocyte migration across the blood-brain barrier (BBB) are therefore regarded as promising therapeutic approaches to combat MS. As the CD40L-CD40 dyad signals via TNF receptor-associated factor 6 (TRAF6) in myeloid cells to induce inflammation and leukocyte trafficking, we explored the hypothesis that specific inhibition of CD40-TRAF6 interactions can ameliorate neuro-inflammation.

**Methods:**

Human monocytes were treated with a small molecule inhibitor (SMI) of CD40-TRAF6 interactions (6877002), and migration capacity across human brain endothelial cells was measured. To test the therapeutic potential of the CD40-TRAF6-blocking SMI under neuro-inflammatory conditions in vivo, Lewis rats and C57BL/6J mice were subjected to acute experimental autoimmune encephalomyelitis (EAE) and treated with SMI 6877002 for 6 days (rats) or 3 weeks (mice).

**Results:**

We here show that a SMI of CD40-TRAF6 interactions (6877002) strongly and dose-dependently reduces trans-endothelial migration of human monocytes. Moreover, upon SMI treatment, monocytes displayed a decreased production of ROS, tumor necrosis factor (TNF), and interleukin (IL)-6, whereas the production of the anti-inflammatory cytokine IL-10 was increased. Disease severity of EAE was reduced upon SMI treatment in rats, but not in mice. However, a significant reduction in monocyte-derived macrophages, but not in T cells, that had infiltrated the CNS was eminent in both models.

**Conclusions:**

Together, our results indicate that SMI-mediated inhibition of the CD40-TRAF6 pathway skews human monocytes towards anti-inflammatory cells with reduced trans-endothelial migration capacity, and is able to reduce CNS-infiltrated monocyte-derived macrophages during neuro-inflammation, but minimally ameliorates EAE disease severity. We therefore conclude that SMI-mediated inhibition of the CD40-TRAF6 pathway may represent a beneficial treatment strategy to reduce monocyte recruitment and macrophage activation in the CNS and has the potential to be used as a co-treatment to combat MS.

**Electronic supplementary material:**

The online version of this article (doi:10.1186/s12974-017-0875-9) contains supplementary material, which is available to authorized users.

## Background

Multiple sclerosis (MS) is a progressive, inflammatory, demyelinating disease of the central nervous system (CNS) that results in the formation of sclerotic plaques in the white and gray matter, causing clinical symptoms, such as weakness, numbness, pain, and visual impairments [1]. Although the etiology of MS remains unknown, the CNS entry of immune cells, especially monocytes and T cells, plays a pivotal role in the development of MS [1–3]. Disruption and inflammation of the blood-brain barrier (BBB) is a prerequisite for leukocyte CNS entry and can be initiated by reactive oxygen species (ROS), matrix metalloproteinases (MMPs), angiogenic factors, inflammatory cytokines, autoantibodies, pathogens, and leukocytes themselves via firm adhesion to brain endothelial cells and subsequent trans-endothelial migration [4]. After BBB passage, monocyte-derived macrophages and T cells promote lesion formation and cause axonal damage [1–3, 5, 6]. In turn, these immune cells secrete pro-inflammatory cytokines and chemokines that promote the recruitment of other immune cells, which amplifies the inflammatory response [1–3, 7]. Strategies that inhibit immune cell migration into the CNS are therefore promising therapeutic approaches to combat MS. The majority of current MS therapies such as anti-very late antigen (VLA)-4 antibodies, MMP inhibitors, interferons, and corticosteroids can successfully reduce the relapse rate as well as the development of new inflammatory CNS lesions that occur after breakdown of the BBB in patients [8–12]. However, a drawback of current therapies is that they target a vital part of the immune system, which induces immune-suppressive side effects [13]. Therefore, there is a high and unmet need for the development of novel and more specific therapeutic strategies.

The co-stimulatory CD40-CD40L dyad has a critical role in the development of immune responses and chronic inflammatory diseases, such as atherosclerosis, obesity, and rheumatoid arthritis [14]. In MS lesions, CD40 is expressed on brain endothelial cells, monocytes, pro-inflammatory (M1) macrophages, astrocytes, and microglia, and CD40L is highly expressed by T cells found in the cerebrospinal fluid of MS patients [15–18]. Exposure of primary human brain microvascular endothelial cells (BMVECs) to soluble CD40L promoted the expression of intercellular adhesion molecule (ICAM)-1 and vascular cell adhesion molecule (VCAM)-1, which led to a fourfold increase in monocyte adhesion to BMVECs [19]. Both *CD40L* ^−/−^ and *CD40* ^−/−^ mice are protected against experimental autoimmune encephalomyelitis (EAE) [15, 18]. CD40 expressed by CNS-endogenous cells is known to control the migration and retention of myelin oligodendrocyte glycoprotein-reactive T cells in the CNS of mice during EAE [20]. Antibody-mediated inhibition of CD40 and CD40L repressed EAE onset and the severity of disease in marmoset monkeys and mice. Moreover, when anti-CD40L antibodies were administered during disease remission in these models, clinical relapses were prevented [21–24]. However, (long-term) antibody-mediated inhibition of CD40L results in thromboembolic events and/or immunosuppression [25]. Specific downstream interference in the CD40L-CD40 pathway is therefore preferable.

Upon binding of CD40L, CD40 recruits tumor necrosis factor receptor-associated factors (TRAFs) to exert signalling [25]. The intracellular domain of CD40 contains a distal binding domain for TRAF2/3/5 and a proximal domain for TRAF6 [25]. Using mice with site-directed mutagenesis for the TRAF6 or TRAF2/3/5 binding site on the CD40 intracellular tail, we demonstrated that CD40-TRAF6 interactions, and not CD40-TRAF2/3/5 interactions, promote the development of atherosclerosis and neointima formation [26, 27]. Mice with a deficiency in CD40-TRAF6 interactions are characterized by decreased numbers of circulating ly6C^high^ monocytes, impaired recruitment of monocytes to the endothelium, and skewing of macrophages towards the anti-inflammatory (M2) profile [26]. To exploit the therapeutic potential of the CD40-TRAF6 axis, we developed small molecule inhibitors (SMIs) of CD40-TRAF6 interactions [28]. SMI 6877002 has been confirmed to have functional specificity for the CD40-TRAF6 and not the CD40-TRAF2/3/5 pathway. The SMI did not show toxicity in an in vitro viability assay or in in vivo treatment [28]. SMI 6877002 was proven to successfully reduce metabolic and inflammatory complications of diet-induced obesity, peritonitis, and sepsis [28, 29].

As the CD40L-CD40 dyad plays a critical role in chronic inflammation and monocyte recruitment and skewing, which are key elements of neuro-inflammation, we here aimed to study the effects of SMI-mediated blockage of the CD40-TRAF6 interaction on human monocyte trans-endothelial migration and activation in vitro*.* In addition, we investigated the effect of our CD40-TRAF6-blocking SMI on neuro-inflammation in vivo*.*

## Methods

### Isolation of human monocytes and treatments

Human blood monocytes were isolated from buffy coats of healthy donors (*n* = 5) (Sanquin blood bank, Amsterdam, The Netherlands, upon written informed consent with regard to scientific use) by Ficoll gradient and CD14-coated beads as described previously [30]. The human brain endothelial cell line hCMEC/D3 [31] was grown in endothelial cell basal medium-2 supplemented with human epidermal growth factor (hEGF), hydrocortisone, GA-1000, fetal bovine serum (FBS), vascular endothelial growth factor (VEGF), human fibroblast growth factor (hFGF-B), R3-insulin-like growth factor (IGF)-1, ascorbic acid, and 2.5% fetal calf serum (EGM-2, Lonza, Basel, Switzerland). Endothelial cells (ECs) were grown to confluence in 96-well plates. Monocytes were incubated with vehicle (DMSO 0.16%) or the small molecule inhibitor (SMI) 6877002 (1–10 μM) [28, 29] for 1 h, after which CD40 signalling was activated using the agonistic CD40 antibody G28.5 (30 μg/ml) combined with IFN-γ (5 ng/ml) for 16 h. In another experiment, monocytes were incubated with G28.5 (30 μg/ml) for 1 h before treatment with SMI 6877002 (1–10 μM) for 16 h. To study the role of ROS in CD40-TRAF6-induced monocyte migration, 50 μM luteolin (a flavonoid with ROS scavenging function, able to inhibit EAE and myelin phagocytosis [32, 33]) (Sigma-Aldrich, St. Louis, MO, USA) was added to the vehicle/SMI-pretreated monocytes 1 h before the migration experiment.

### In vitro trans-endothelial migration assay

We used two established protocols for the measurement of human monocyte migration across brain endothelial cells using a Transwell system [34] and/or time-lapse video microscopy [35] with minor modifications. For Transwell migration experiments, we used a Transwell system (Costar, Corning, Amsterdam, The Netherlands) with polycarbonate filter pore size of 5 μm, which were coated with collagen type 1 (Sigma-Aldrich, Zwijndrecht, The Netherlands). The hCEMC/D3 cells were seeded at a concentration of 1 × 10^4^ cells per well in endothelial cell basal medium-2 (Lonza) supplemented with 2.5% FCS (Lonza) and were cultured to confluent monolayers. After extensive washing, monocytes were re-counted and suspended in culture medium (7.5 × 10^5^ cells/ml) and were added to brain endothelium monolayers and incubated for 8 h. To determine the number of migrated cells, trans-migrated cells were transferred to FACS tubes, and 20,000 beads (Beckman Coulter, USA) were added to each sample. Samples were analysed using a FACSCalibur (Becton Dickinson, Belgium), and the number of migrated monocytes was determined based on 5000 gated beads. The absolute number of migrated monocytes is presented compared to the total number of monocytes added to the upper chamber as described [36].

For time-lapse video microscopy experiments, monocytes were added to brain endothelial monolayers and the number of migrated monocytes was assessed after 4 h using an inverted phase-contrast microscope (×40 magnification, Nikon Eclipse TE300) housed in a temperature-controlled (37 °C), 5% CO_2_ gassed chamber (manufactured for this purpose). A field of 200 μm^2^ was randomly selected and recorded for 10 min at 50 times normal speed using a color video 3CCD camera (Sony, using a CMAD2 adapter) coupled to a time-lapse video recorder (Sony SVT S3050P). After recording, tapes were replayed at normal speed and analysed by enumerating the number of cells within the field that had migrated through the monolayer. All experiments were performed in triplicate with at least three different donors.

### Dihydrorhodamine assay

ROS production by monocytes was measured using dihydrorhodamine (DHR) (Sigma-Aldrich, Munich, Germany), which reacts with ROS in a peroxidase-like reaction to yield fluorescent rhodamine 123 [37]. After incubation with the SMI and stimulation with the agonistic CD40 antibody G28.5 as described above, cells were rinsed twice with RPMI, re-counted (7.5 × 10^5^ cells/ml) and incubated for 30 min at 37 °C with 0.5 μM DHR in RPMI medium. After that, cells were rinsed twice with PBS/BSA 0.1% and transferred to FACS tubes. Analysis of cells fluorescent for rhodamine 123 was performed by flow cytometry with excitation at 488 nm and the emitted fluorescence collected at 525 nm.

### Analysis of cytokine profiles

The production of pro- and anti-inflammatory mediators was assessed by enzyme-linked immunosorbent assay (ELISA) in cell-free supernatants of vehicle- or SMI-treated CD40-stimulated monocytes using commercial kits for human IL-10, IL-6, and TNF-α CytoSet ELISA kit (Biosource, Nivelles, Belgium) according to the manufacturer’s protocol. The samples were measured using a Luminex 200 (Bio-Rad, Hercules, CA, USA).

### Flow cytometry

SMI-treated and untreated human monocytes were incubated with primary antibody (50 mg/ml Nanogam, Sanquin, The Netherlands) diluted in FACS buffer (PBS containing 0.5% bovine serum albumin (BSA) and 2 mM EDTA) to prevent non-specific binding of antibodies to the Fc receptors. Cells were then incubated with fluorescently labeled secondary antibodies CD14, CD16, HLA-DR, CD80 (BD, Breda, The Netherlands), and CD86 (BioLegend, San Diego, CA, USA), and staining was analysed by flow cytometry (FACSCanto II, BD Biosciences, Breda, The Netherlands) and FlowJo software version 7.6.5 (Tree Star).

### EAE induction rats

Eight-week-old male Lewis rats were obtained from Harlan and maintained at the animal facility of the VU University Medical Center. The animals had ad libitum access to food and water and were housed under a 12-h light/dark cycle.

To induce EAE, rats were injected subcutaneously with 20 μg myelin basic protein (MBP) isolated from guinea pig brain and spinal cord (Harlan Laboratories, Horst, The Netherlands) in PBS mixed with complete Freund’s adjuvant (CFA; 4 mg/ml *Mycobacterium tuberculosis* H37Ra; Difco Laboratories, Detroit, MI, USA). A control group without EAE induction was included (*n* = 6). EAE animals were treated by intraperitoneal (i.p.) injection with the vehicle (0.05% Tween 80, 2% DMSO in saline) (*n* = 11) or with 10 μmol/kg SMI 6877002 (*n* = 11) from days 6 to 11 after the induction of EAE. This phase of the disease is mainly characterized by the recruitment of inflammatory cells to the CNS. Neurological symptoms were scored daily and graded from 0 to 5: 0 = no neurological abnormalities; 0.5 = partial loss of tail tonus; 1 = complete loss of tail tonus; 2 = hind limb paresis; 3 = hind limb paralysis; 4 = paralysis up to the diaphragm; 5 = death. Body weight was measured daily. Animals were sacrificed 14 and 20 days after induction of EAE. All the experimental procedures were approved by the Ethical Committee for Animal Experiments of the VU University Medical Center (VUMC). Scoring of clinical symptoms was performed by an observer who was blinded to the experimental conditions.

### Histology and immunohistochemistry of rat cerebellum and spinal cord

The brain was collected, snap-frozen in liquid nitrogen, and stored at −80 °C. The spinal cord was fixed in 4% paraformaldehyde and embedded in paraffin. Inflammation of the spinal cord was graded on 4-μm haematoxylin-eosin (H&E)-stained sections. Immunohistochemistry on the spinal cord was performed for CD68 (1:200, polyclonal, Abcam Inc., Cambridge, MA, USA) and for CD3 (1:200, clone G4.18, eBioscience, San Diego, CA, USA). The cerebellum was embedded in Tissue Tek, and 6-μm sections were used for staining with rabbit anti-laminin (1:200, clone 6e3, EY Laboratories, San Mateo, USA) to localize CNS infiltrates, ED1 (1:100, AbD Serotec, Puchheim, Germany) to detect macrophages, or R7.3 (1:85, BD Biosciences, San Jose, CA, USA) to detect T cells. Nuclei were visualized by DAPI (Invitrogen, Eugene, USA). All other organs were analysed following H&E staining. Analyses were performed by an observer who was blinded to the experimental conditions.

### RNA isolation and qPCR of rat spinal cord

Total RNA was extracted from the spinal cord using TRIzol (Invitrogen, Carlsbad, CA, USA) and reverse-transcribed using an iScript cDNA synthesis kit (Bio-Rad, Veenendaal, The Netherlands). Quantitative (q)PCR was performed with a SYBR Green PCR kit (Applied Biosystems, Leusden, The Netherlands) on a ViiA7 real-time PCR system (Applied Biosystems, Leusden, The Netherlands). Expression levels of transcripts obtained with real-time PCR were normalized to GAPDH expression levels. The following rat primers were used: GAPDH FW: 5′-AGGTTGTCTCCTGTGACTTC-3′, GAPDH RV: 5′-CTGTTGCTGTAGCCATATTC-3′, CD40 FW: CD40 RV: 5′-CTTAACCTGAAGCCCTTGATTG-3′, CD80 5′-TTCCACGTCTCAGGTTCATTC-3′, CD80 RV: 5′-GTAATCACAGGACAGCAATGC-3′, CD86 FW: 5′-TCTGTGCTGTCTCTTTCTGC-3′, CD86 RV: 5′-TTGATCGACTCGTCAACACC-3′, TNF FW: 5′-CTTCTCATTCCTGCTCGTGG-3′, TNF RV: 5′-TGATCTGAGTGTGAGGGTCTG-3′, NOS2 FW: 5′-GGAGCAGGTTGAGGATTACTTC-3′, NOS2 RV: 5′-TCAGAGTCTTGTGCCTTTGG-3′, MMP2 FW: 5′-AGGGCACCTCTTACAACAGC-3′, MMP2 RV: 5′-CCCGGTCATAATCCTCGGTG-3′, MMP9 FW: 5′-GATCCCCAGAGCGTTACTCG-3′, MMP9 RV: 5′-GTTGTGGAAACTCACACGCC-3′.

### EAE induction mice

To investigate the effects of extended 6877002 treatment in EAE, a second model was used. Ten-week-old female C57BL/6J mice were obtained from Charles River Laboratories and maintained at the animal facility of the Academic Medical Center, Amsterdam. The animals had ad libitum access to food and water and were housed under a 12-h light/dark cycle. They were treated daily by i.p. injection with the vehicle (0.05% Tween 80, 2% DMSO in saline) (*n* = 14) or with 10 μmol/kg SMI 6877002 (*n* = 14) starting 3 days before EAE induction until 17 days after the induction of EAE. On day 0, mice were immunized subcutaneously with 200 μg of a myelin oligodendrocyte glycoprotein peptide (MOG_35-55_) emulsified in CFA supplemented with 4 mg/ml *M. tuberculosis* H37Ra (Hooke Laboratories, Lawrence, MA, USA). Mice were injected i.p. on days 0 and 1 with 400 ng pertussis toxin. A control group without EAE induction was included (*n* = 6). Neurological symptoms were monitored daily using the grading scale as follows: 0 = no neurological abnormalities; 0.5 = partial loss of tail tonus; 1 = complete loss of tail tonus; 2 = hind limb paresis; 3 = partial hind limb paralysis; 4 = complete hind limb paralysis; 4.5 = paralysis up to the diaphragm, 5 = death. Body weight was measured daily, and the animals were sacrificed 17 days after induction of EAE. All the experimental procedures were approved by the Ethical Committee for Animal Experiments of the Academic Medical Center, Amsterdam (AMC). Scoring of clinical symptoms was performed by an observer who was blinded to the experimental conditions.

### Flow Cytometry

Blood was obtained by cardiac puncture and collected using EDTA-filled syringes. The spleen and lymph nodes were collected. Erythrocytes in the blood and spleen were removed by incubation with hypotonic lysis buffer (8.4 g of NH_4_Cl and 0.84 g of NaHCO_3_ per litre of distilled water). To prevent non-specific binding of antibodies to the Fc receptor, all cell suspensions were incubated with a CD16/32 antibody (eBioscience, San Diego, CA, USA) prior to labelling. CD45, CD19, CD8, FoxP3 (eBioscience, San Diego, CA, USA), CD3 (BioLegend, San Diego, CA, USA), CD11b, and CD4 (BD, Breda, The Netherlands) antibodies were incubated with the indicated tissues. Staining was analysed by flow cytometry (FACSCanto II, BD Biosciences, Breda, The Netherlands) and FlowJo software version 7.6.5 (Tree Star).

### Histology and immunohistochemistry of the mouse cerebellum

The cerebellum was collected and fixed in 4% paraformaldehyde and embedded in paraffin. Inflammation was graded on 4-μm-thick H&E-stained sections. Immunohistochemistry was performed for Mac3 (BD, Breda, The Netherlands) and CD3 (AbD Serotec, Puchheim, Germany). Per section, 8–12 pictures were taken to include the complete cerebellum surface and analysed by an observer who was blinded to the experimental conditions.

### RNA isolation and qPCR of mice spinal cord

RNA isolation of the spinal cord, cDNA synthesis, and qPCR were performed as described above. Expression levels of transcripts obtained with real-time PCR were normalized to the mean expression levels of the three housekeeping genes GAPDH, CycloA, and Rplp0. The following mouse primers were used: GAPDH FW: 5′-CAACTCACTCAAGATTGTCAGCAA-3′, GAPDH RV: 5′-TGGCAGTGATGGCATGGA-3′, CycloA FW: 5′-TTCCTCCTTTCACAGAATTATTCCA-3′, CycloA RV: 5′-CCGCCAGTGCCATTATGG-3′, Rplp0 FW: 5′-GGACCCGAGAAGACCTCCTT-3′, Rplp0 RV: 5′-GCACATCACTCAGAATTTCAATGG-3′, IFN-γ FW: 5′-GAGGAACTGGCAAAAGGATGG-3′, IFN-γ RV: 5′-TGTTGCTGATGGCCTGATTG-3′, IL-17 FW: 5′-TCCCTCTGTGATCTGGGAAG-3′, IL-17 RV: 5′-CTCGACCCTGAAAGTGAAGG-3′, FoxP3 FW: 5′-CCCAGGAAAGACAGCAACCTT-3′, FoxP3 RV: 5′-TTCTCACAACCAGGCCACTTG-3′. TNF FW: 5′-CATCTTCTCAAAATTCGAGTGACAA-3′, TNF RV: 5′-TGGGAGTAGACAAGGTACAACCC-3′, IL-10 FW: 5′-TTTGAATTCCCTGGGTGAGAA-3′, IL-10 RV: 5′-CTCCACTGCCTTGCTCTTATTTTC-3′, MCP-1 FW: 5′-AGCACCAGCCAACTCTCACT-3′, and MCP-1 RV: 5′-CGTTAACTGCATCTGGCTGA-3′.

### Statistical analysis

Results are presented as mean ± SEM. Data were analysed by Student’s *t* test, clinical EAE scores were analysed by ANOVA and Bonferroni post-tests, and the clinical parameters were analysed by a non-parametric (Mann-Whitney) test. The log-rank test was used for survival analysis. Calculations were performed using GraphPad Prism 5.0 software (GraphPad Software, Inc., La Jolla, CA, USA). *P* values <0.05 were considered statistically significant.

## Results

### Inhibition of CD40-TRAF6 interactions by SMI 6877002 reduces trans-endothelial migration of human monocytes and ROS production by these cells

**Fig. 1:**
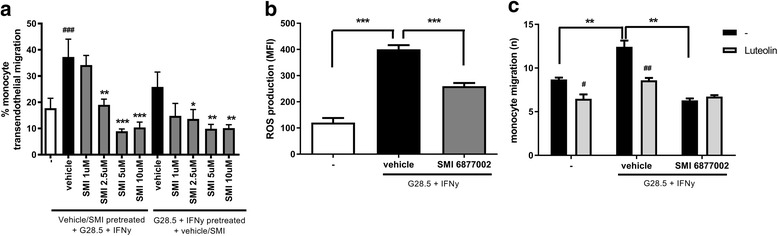
SMI treatment of monocytes inhibits CD40-induced trans-endothelial migration by limiting ROS production. Human monocytes were treated with either SMI 6877002 (1–10 μM) or vehicle for 1 h and stimulated with G28.5 (agonistic CD40 antibody) for 16 h, or pretreated with G28.5 for 1 h and then stimulated with SMI 6877002 for 16 h. **a** Monocyte trans-endothelial migration was studied in vitro using hCMEC/D3 cells by Transwell migration [31]. **b** ROS production measured as mean fluorescence intensity (*MFI*) of rhodamine 123. **c** CD40-induced monocyte migration in the presence or absence of the ROS scavenger luteolin (50 μM), measured in time-lapse migration. Experiments were performed in triplicate using buffy coats from 3 (1A), 4 (1B), or 3 (1C) human donors, and results are presented as the mean ± SEM. */#*P* < 0.05, **/##*P* < 0.01,***/###*P* < 0.001 as determined by Student’s *t* test. #luteolin vs control

As migration of inflammatory cells across the BBB represents a pathological hallmark of MS, we analysed the effects of the CD40-TRAF6-blocking SMI on monocyte migration across an in vitro BBB [31]. Activation of CD40 signalling in monocytes using the agonistic CD40 antibody G28.5 and IFN-γ increased their trans-endothelial migration across non-activated EC by 210% (1 h vehicle pretreated) or 146% (Fig. 1a). When monocytes were treated with the SMI (before or after CD40 activation), a dose-dependent reduction in trans-endothelial migration was observed (Fig. 1a). In contrast, SMI treatment of brain endothelial cells had no effect on monocyte trans-endothelial migration (data not shown), suggesting that the SMI specifically affects CD40 on monocytes and does not block CD40 signalling in endothelial cells. Cell viability was unaffected by the SMI treatment (data not shown).

Reactive oxygen species play an important role in neurodegenerative diseases like MS. Pro-inflammatory mediators and oxidizing radicals are produced by adherent monocytes, infiltrating macrophages and activated microglia [38]. These locally generated ROS induce BBB disruption and enhance leukocyte migration in the initial phase of MS lesion formation [39]. To assess whether SMI treatment affects ROS production by human monocytes, we activated CD40 in the presence or absence of the CD40-TRAF6-blocking SMI and measured ROS production. As shown in Fig. 1b, CD40-induced ROS production by monocytes was significantly reduced by treatment with SMI 6877002 (35.1%). To address whether the inhibiting effects of SMI 6877002 on monocyte migration were ROS dependent, we introduced the flavonoid luteolin in our in vitro BBB system. Luteolin decreased the trans-endothelial migration capacity of non-treated monocytes, as described before [33]. Notably, CD40-induced monocyte migration was blocked when these monocytes were treated with luteolin, revealing an important role for ROS in CD40-induced monocyte trans-endothelial migration (Fig. 1c). Interestingly, luteolin had no effect on the migration of SMI-treated monocytes, which is in line with our assumption that both have a similar mechanism, which is inhibition of ROS production.

Together, these data indicate that SMI-mediated inhibition of CD40-TRAF6 interactions in monocytes impairs the recruitment of these cells, to some extent in a ROS-dependent manner.

### Small molecule inhibitors of the CD40-TRAF6 interaction reduce inflammation of human monocytes

**Fig. 2:**
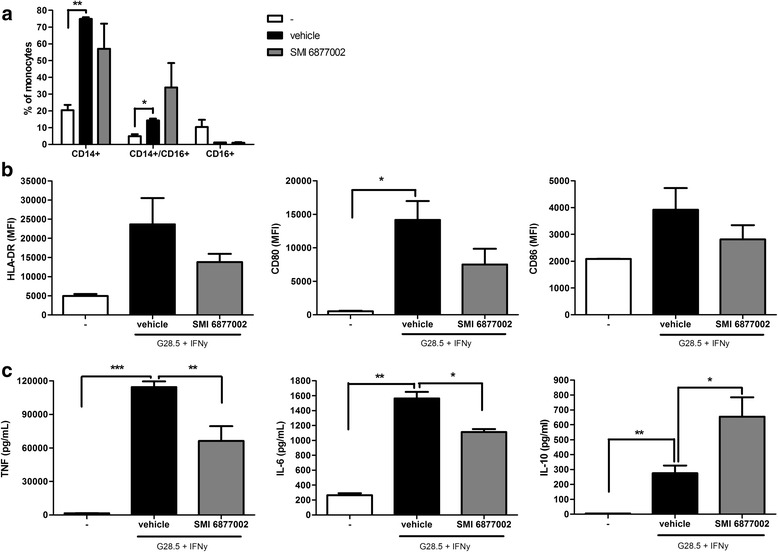
CD40-TRAF6-inhibiting SMI 6877002 improved the inflammatory phenotype of monocytes. Human monocytes were treated with SMI 6877002 (2.5 μM) for 1 h and stimulated with G28.5 (agonistic CD40 antibody) for 16 h. **a** CD14^+^ (classical), CD14^+^/CD16^+^ (intermediate), and CD16^+^ (non-classical) subsets of monocytes were measured by flow cytometry. **b** HLA-DR, CD80, and CD86 mean fluorescent intensity (*MFI*) measured in the classical monocyte subset (CD14^+^) by flow cytometry. **c** TNF, IL-6, and IL-10 production were measured by ELISA. Experiments were performed in triplicate using buffy coats of 2 (flow cytometry) or 4 (ELISA) human donors, and results are presented as the mean ± SEM. *P < 0.05, **P < 0.01, ***P < 0.001 as determined by Student’s *t* test

Human monocytes can be divided into a classical, CD14^+^ pro-inflammatory subset, a non-classical CD16^+^ subset, and an intermediate subset positive for both CD14 and CD16 [35]. To assess whether SMI treatment affects the inflammatory phenotype of monocytes, we performed flow cytometry on the cells and ELISA on the supernatant. SMI treatment results in a relative smaller subset of CD14^+^ monocytes and more intermediate CD14^+^/CD16^+^ monocytes (Fig. 2a). Moreover, SMI treatment results in trends towards reduced HLA-DR, CD80, and CD86 expression in the classical monocyte subset (CD14^+^) compared to the vehicle-treated monocytes (Fig. 2b). Besides affecting ROS production, SMI 6877002 was also able to reduce CD40-induced TNF production in human monocytes, both on the protein level (Fig. 2c) and the transcript level (12.4-fold increase in vehicle-treated monocytes vs 7.4-fold increase in SMI-treated monocytes compared to untreated cells, data not shown). Moreover, SMI treatment reduced IL-6 levels and increased the levels of the anti-inflammatory cytokine IL-10 (Fig. 2c).

Collectively, these data indicate that our CD40-TRAF6-inhibiting SMI is capable of antagonizing the CD40-induced pro-inflammatory profile of monocytes, and increasing IL-10 production, thereby generating a more anti-inflammatory monocyte phenotype, less capable of traversing the brain endothelial barrier in vitro.

### SMI 6877002 treatment ameliorates EAE in rats

To study the effects of our SMI on neuro-inflammation in vivo, we induced acute EAE in rats and treated them daily from days 6 to 12 with 10 μM/kg SMI 6877002, or vehicle. All EAE-induced rats developed clinical symptoms of EAE and none of the animals died due to EAE (Table 1). Body weight was not affected by the treatment (Table 1), and haematoxylin and eosin staining of the spleen, liver, heart, lung, gastrointestinal tract, kidney, bladder, and lymph nodes revealed no toxic, immunosuppressive, or thromboembolic side effects of the SMI.

**Table 1:** Clinical parameters of rats subjected to EAE and treated with vehicle or SMI 6877002

Clinical parameter	Vehicle	SMI 6877002	Statistics
Incidence (%)	100	100	
Survival (%)	100	100	
AUC	10.6	8.7	*P* = 0.0804
Mean clinical score (day 13)	2.9	2.0	*P* = 0.1160
Mean day of onset	11.1	11.5	*P* = 0.4740
Mean peak disease severity	3.4	2.9	*P* = 0.0184
% Body weight loss (day 12 compared to day 0)	5.4	4.7	*P* = 0.2636

Treatment with SMI 6877002 reduced the cumulative score (AUC), the mean clinical score on day 13, and the peak disease severity compared to vehicle-treated rats. *P* values <0.05 were considered statistically significant, as determined by the non-parametric Mann-Whitney *U* test for disease scores or Student’s *t* test for body weight

**Fig. 3:**
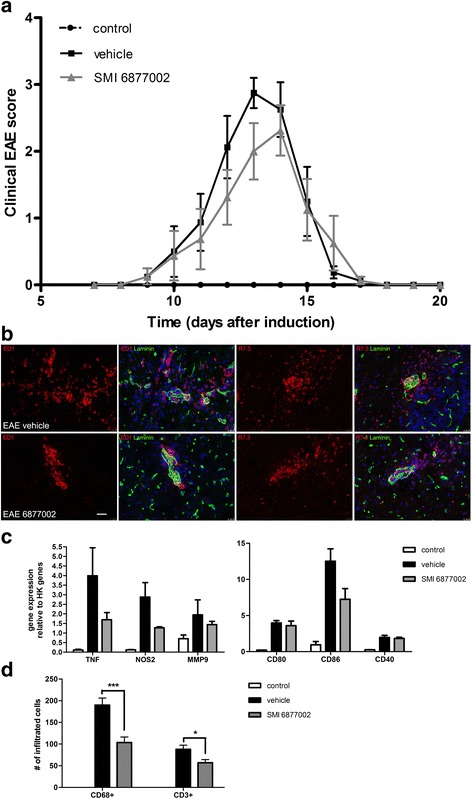
SMI 6877002 treatment ameliorates severe paralysis in rats subjected to EAE. **a** EAE was induced, and animals were treated with 10 μmol/kg SMI 6877002 or vehicle from days 6 to 11 after induction. Clinical scores were observed daily. Experiments were performed with 6 animals in the control group and 11 animals in the EAE and SMI groups. **b** Immunofluorescence analysis of rat EAE cerebellum to determine macrophage and T cell infiltration into the CNS parenchyma. Sections were stained for ED1 (in *red* for macrophages), R7.3 (in *red* for T cells), and laminin (in *green* for localization). Representative images from three animals per group sacrificed at the peak of the disease. *Scale bar* 25 μm. **c** Gene expression in rat spinal cord during peak of disease was measured by qPCR. mRNA expression levels of *TNF*, *NOS2*, *MMP9*, *CD80*, *CD86*, and *CD40* presented as relative expression compared to *GAPDH*. Expression was measured in three animals per group. **d** Quantified numbers of CD68 (for macrophages)- and CD3 (for T cells)-positive immune cell infiltrates in spinal cord tissues collected at day 20 of EAE. For each animal, the amount of infiltrates was counted on four levels, 5 mm between sections. Results are presented as the mean ± SEM. *P < 0.05, ***P < 0.001 as determined by Student’s *t* test

The peak disease severity was significantly reduced in rats treated with SMI 6877002 compared to vehicle-treated rats, and the cumulative score (AUC) was smaller, but not significantly reduced, in the SMI-treated rats (Fig. 3a and Table 1). The SMI treatment had no significant effect on the day of onset of EAE symptoms.

These in vivo findings suggest that SMI 6877002 is able to ameliorate the severity of EAE.

### Blocking CD40-TRAF6 interactions limits macrophage influx into the cerebellum of EAE-induced rats and reduces inflammation in the spinal cord

To determine the phenotype and localization of CNS infiltrates during EAE, we performed immunohistochemistry on the cerebellum of three rats sacrificed at the peak of disease (day 14 after EAE induction). Upon SMI treatment, we observed reduced numbers of macrophages and/or activated microglia (ED1^+^ cells) in white matter lesions and we detected accumulation of monocyte-derived macrophages in the perivascular spaces whereas in vehicle-treated EAE animals, macrophages and/or activated microglia were predominantly present in the brain parenchyma (Fig. 3b). SMI treatment did not affect the localization or amount of T cells (R7.3^+^ cells) as these cells were found in both the perivascular spaces and white matter lesions in all groups (Fig. 3b). Accordingly, transcript levels of TNF, nitric oxide synthase (NOS2), MMP9, and CD86 in the spinal cord at the peak of the disease (*n* = 3 per group) showed a slight, but not significant, reduction (Fig. 3c). Further analysis of the spinal cords obtained from the rats sacrificed *after* recovery of EAE revealed similar findings. The numbers of macrophages and/or activated microglia (CD68^+^ cells) and T cell (CD3^+^ cells) accumulation in the spinal cord after recovery of EAE were significantly lower in the SMI-treated group compared to the non-treated controls (Fig. 3d), and transcript levels of TNF, NOS2, and MMP9 were reduced (Additional file 1: Figure S1).

Thus, the ability of SMI 6877002 to reduce the number of macrophages and/or activated microglia in the brain parenchyma and to diminish gene expression of pro-inflammatory markers in the spinal cord may explain the observed decrease in the severity of clinical signs in the SMI-treated animals compared to untreated EAE animals.

### Blocking CD40-TRAF6 interactions does not ameliorate EAE in mice, but decreases macrophage accumulation in the CNS

**Fig. 4:**
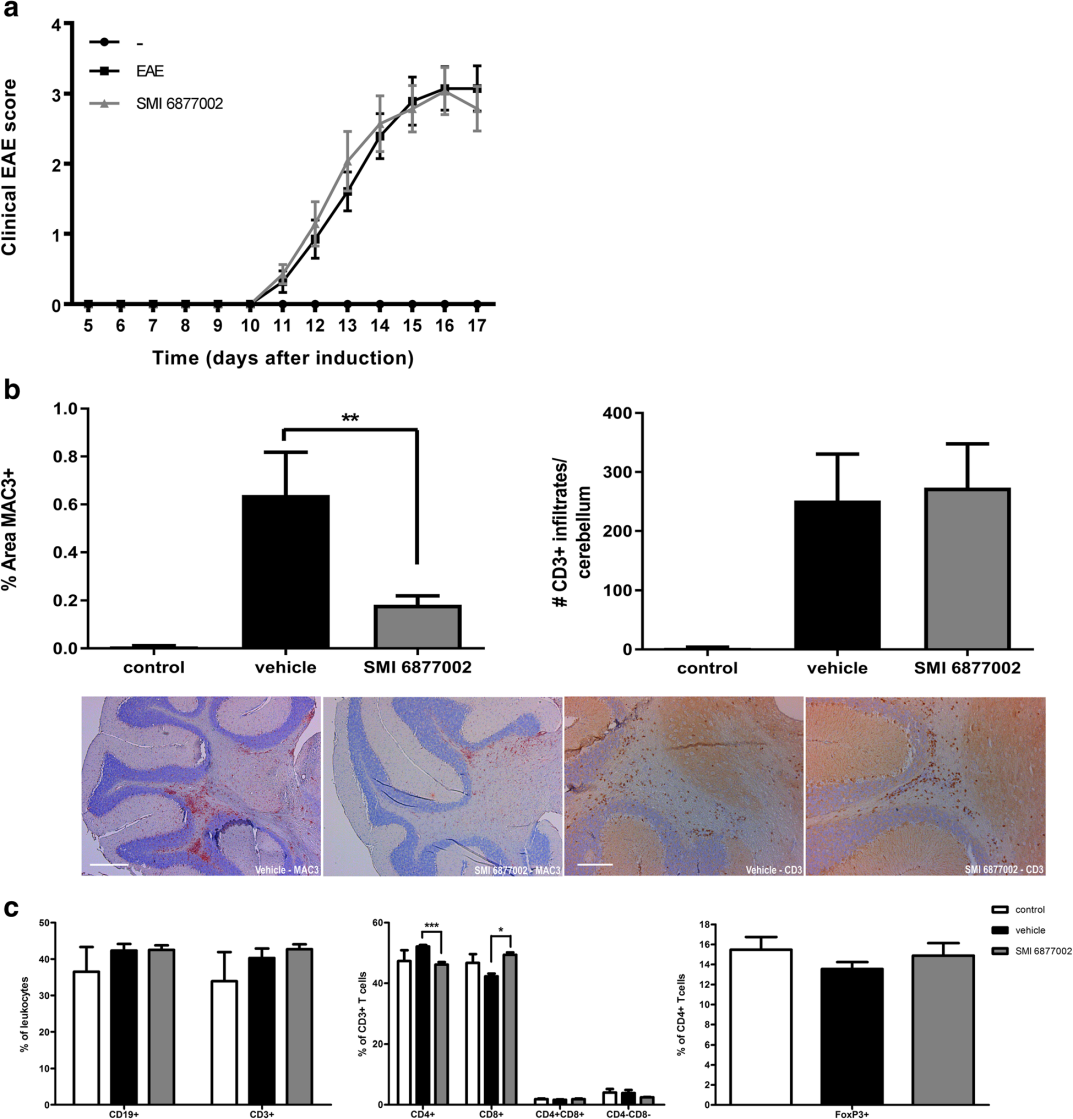
Prolonged SMI treatment does not affect EAE development in mice. Mice were treated with vehicle or 10 μmol/kg SMI 6877002 starting 3 days prior to EAE induction until 17 days after immunization. **a** Clinical scores of mice treated with vehicle or SMI 6877002. Experiments were performed with 14 animals in the vehicle- and SMI-treated groups and 6 control animals without EAE induction. **b** SMI 6877002 treatment reduces the percentage of Mac3^+^ cells in the cerebellum of EAE mice at the peak of disease (*scale bar* Mac3 staining 400 μm), but has no effect on T cell infiltration into the cerebellum (*scale bar* CD3 staining 200 μm). **c** Flow cytometric analysis demonstrates that SMI 6877002 treatment results in a shift in the CD4/CD8 T cell balance in lymph nodes. Analysis was performed in lymph nodes of six animals per group sacrificed at the peak of disease. Results are presented as the mean **±** SEM. *P < 0.05, ***P < 0.001 as determined by Student’s *t* test

To confirm the protective effect of SMI 6877002 in a different model of EAE, EAE was induced in C57BL/6J mice. In this model, mice were treated with the SMI or vehicle starting 3 days before EAE induction until sacrifice at the peak of clinical symptoms. Body weight was not significantly affected by the treatment (Table 2). Although SMI-treated mice had a slightly better survival compared to vehicle-treated mice, SMI treatment had no effect on disease severity or day of onset of disease in this model (Fig. 4a and Table 2).

**Table 2:** Clinical parameters of mice subjected to EAE and treated with vehicle or SMI 6877002

Clinical parameter	Vehicle	SMI 6877002	Statistics
Incidence (%)	93.3	100.0	
Survival (%)	86.7	100	*P* = 0.1641
AUC	12.75	13.39	*P* = 0.3461
Mean clinical score (day 15)	2.9	2.8	*P* = 0.9257
Mean day of onset	12.1	12.6	*P* = 0.6487
Mean peak disease severity	3.4	3.3	*P* = 0.8853
% Body weight loss (day 16 compared to day 0)	12.2	12.1	*P* = 0.6548

Treatment with SMI 6877002 has no significant effect on clinical parameters of EAE in mice. *P* values <0.05 were considered statistically significant, as determined by the log-rank test for survival, the non-parametric Mann-Whitney *U* test for disease scores, or Student’s *t* test for body weight

However, in the cerebellum of the SMI 6877002-treated mice, we observed a significant reduction in numbers of Mac3^+^-stained cells compared to the vehicle-treated mice (Fig. 4b), indicating reduced macrophage accumulation and/or microglia activity. There was no difference in the number of CD3^+^ cells infiltrated in the cerebellum (Fig. 4b), showing that, in line with the rat experiments, the SMI inhibits macrophage infiltration, but not T cell infiltration, into the CNS parenchyma.

Flow cytometry on the lymph nodes (LN) of the SMI- and vehicle-treated animals subsequently showed no difference between the groups in the total T cell percentage (CD3^+^) of the leukocyte population, but we observed a change in CD4/CD8 T cell balance. CD4^+^ T cells are found to be more important in the induction of EAE through their production of IL-17, while CD8^+^ T cells express higher levels of the suppressive cytokine IL-10 and have a more regulatory role in the later stage of EAE [40]. SMI-treated mice had proportionally less CD4^+^ T helper cells and more CD8^+^ cytotoxic T cells in the lymph nodes compared to vehicle-treated mice (Fig. 4c), suggesting a shift from EAE-inducing T cells towards more suppressive T cells. The percentage of regulatory T cells (FoxP3^+^) was not affected by the SMI treatment (Fig. 4c). Analysis of immune cell subsets in the blood and spleen showed no effect of SMI treatment in EAE mice on circulating leukocytes (Additional file 2: Figure S2a,b).

The spinal cords of the mice sacrificed at the peak of EAE were used for gene expression analysis. mRNA expression of IL-10, TNF, MCP1, IFN-γ, IL-17, and FoxP3 in the spinal cord did not differ between the groups (Additional file 3: Figure S3).

Taken together, these data show that our CD40-TRAF6-blocking SMI predominantly impairs monocyte and macrophage recruitment into the CNS, reduces neuro-inflammation, and can decrease disease severity in a rat model of EAE and improve survival in a mouse model of EAE. Moreover, SMI treatment can direct the T cell phenotype in the lymph nodes towards a more EAE-protective CD8^+^ subtype.

## Discussion

Antibody-mediated inhibition of CD40L or CD40 is able to reduce the severity of inflammatory diseases. Patients involved in phase I/II trials who received anti-CD40L-blocking therapy for proliferative lupus glomerulonephritis, multiple myeloma, non-Hodgkin’s lymphoma, and systemic lupus erythematosus showed clinical improvement [41–45]. However, all clinical trials were halted after the report that anti-CD40L treatment bears the risk of the development of thromboembolic events [46, 47]. After a successful pilot study with anti-CD40L mAb (IDEC-131) in 15 MS patients (treatment with anti-CD40L revealed a profound reduction in clinical relapse rate in relapsing-remitting MS), a phase II trial with 46 MS patients was launched by Lloyd Kasper and Randolph Noelle in 2002 but was halted soon after a case of severe thromboembolism occurred in a similar trial in Crohn’s disease patients [48, 49]. Another risk of anti-CD40L or anti-CD40 therapy is the development of immune-suppressive side effects [50].

To circumvent these complications, specific downstream interference in the CD40L-CD40 pathway is preferable. Therefore, in this study, we suppressed the CD40-CD40L dyad with a small molecule inhibitor that was generated to target the interaction between CD40 and TRAF6 and leave CD40-TRAF2/3/5 interactions intact [28]. SMI 6877002 was designed using a structure-based virtual ligand screen [28, 29] and has been shown to be an efficient and specific inhibitor of CD40-TRAF6 interactions in mice both in vitro and in vivo [28]. This SMI has proven to reduce adipose tissue inflammation, improve insulin resistance in diet-induced obesity, and reduce peritonitis and polymicrobial sepsis in mice [28, 29].

CD40 is known to be an activator of ROS production [51], and ROS is a strong driver of monocyte recruitment [39]. By treating monocytes with superoxide, van der Goes and colleagues could show that ROS play a key role in driving monocyte adhesion and trans-migration across endothelial cells [39]. Here we showed that the CD40-TRAF6-inhibiting SMI prevents ROS production by human monocytes and thereby decreases their trans-endothelial migration capacity.

In addition, SMI-treated monocytes produced less TNF upon CD40 activation. TNF is well known for its properties to activate brain endothelium and to increase vascular permeability of the BBB, leading to leukocyte trans-endothelial migration, entry of antigens, and activation of microglia [4]. Not only did our SMI prevent the trans-endothelial migration capacity of monocytes, but the SMI also changed the phenotype of the monocyte itself towards a more anti-inflammatory cell type with less CD14^+^ monocytes, reduced HLA-DR, CD80, and CD86 expression, and increased production of IL-10. Skewing of the monocyte phenotype towards a less inflammatory profile upon SMI treatment together with the ability of the SMIs to reduce migration of monocytes across the BBB is what may ultimately lead to a reduction in inflammatory lesions and/or axonal damage in MS patients.

It was previously shown that both *CD40L* ^−/−^ and *CD40* ^−/−^ mice are protected against experimental autoimmune encephalomyelitis (EAE) [15, 18]. In addition to this, anti-CD40L antibody treatment, when administered to mice at the time of EAE induction, blocked the development of acute disease. Treatment at the peak of acute disease resulted in a marked reduction in the relapse rate with fewer mice exhibiting clinical signs of relapse in the anti-CD40L antibody-treated group [24]. Treatment with anti-CD40L mAb around day 6 and day 9 after EAE induction still resulted in blockade of disease by 80 and 67%, respectively, as compared with the complete inhibition (100%) in animals treated with anti-CD40L mAb around day 2 [18].

To study the effects of our SMI on neuro-inflammation in vivo, we used the EAE animal model for MS in Lewis rats and C57BL/6J mice. Upon induction of EAE, we demonstrated that rats treated with SMI 6877002 had significant reduced disease severity compared to vehicle-treated rats. The rats were treated with the SMI starting 6 days after induction of EAE and not at the induction of EAE. We selected this moment based on our in vitro findings in the BBB model, showing that our SMI is able to reduce monocyte migration and activation. As the target of our treatment was the later-occurring activation and migration of monocytes instead of the early activation of T cells, we started the treatment after EAE induction. SMI treatment resulted in modest reduction in clinical symptoms, comparable to what Howard et al. and Gerritse and colleagues found in mice when using an anti-CD40L antibody 6–9 days after induction of EAE [18, 23, 24]. Interestingly, after SMI treatment, we were able to show that monocyte-derived macrophages did not enter the CNS parenchyma as normally seen during EAE, but stay ‘trapped’ in the perivascular space. These findings are in accordance with research of Laman and co-workers, as they showed that in the CNS of anti-CD40 mAb-treated marmoset monkeys with EAE, most infiltrates were found in the perivascular space and only occasionally in the parenchyma [21]. Owen and co-workers further showed that when immune cells stay trapped in the CNS parenchyma, EAE did not occur [52]. Quantification of immune cells in spinal cord tissues revealed less CD68- and CD3-positive accumulated cells in SMI 6877002-treated rats compared to the vehicle-treated EAE animals after recovery of EAE. These results are in accordance with our in vitro data that showed that SMI treatment of monocytes affects their migration capacity. The outcomes of our study with SMI- and vehicle-treated rats are in line with previous studies using anti-CD40L-blocking antibodies as this resulted in a reduction in spinal cord cell infiltration and inflammation and prevented demyelination [23]. SMI 6877002 treatment of EAE rats in the present study resulted in reduced severity of clinical EAE symptoms, which is most likely explained by reduced monocyte migration into the CNS parenchyma. In mice treated with our SMI, no effects on clinical parameters were observed, although macrophage infiltration into the CNS parenchyma was reduced as well.

A possible explanation for the mild effects observed with SMI 6877002 in EAE could be the partial blockade of the CD40 signalling. Our SMI only blocks the CD40-TRAF6 pathway and leaves the CD40-TRAF2/3/5 signalling intact. This is preferable to minimize immune-suppressive side effects, but the CD40-TRAF2/3/5 signalling pathway might be compensating for the loss of CD40-TRAF6 signalling, and the function of only one of the CD40 signalling pathways could be sufficient for CD40 signalling in EAE.

Another explanation may be found in the specificity of our SMI for monocytes and macrophages. Although macrophages play a major role in EAE, EAE is also a T-cell driven disease. Here we show that our SMI is able to interfere with monocyte/macrophage transendothelial migration, but is not sufficient to strongly decrease disease severity, suggesting that the T-cell component is still causing disease symptoms. It may therefore be interesting to use the SMI, targeting monocytes and macrophages, in co-treatment with T cell-targeting drugs, such as interferons, in MS.

Another approach is to think of improvements in our SMI to target the T cell-mediated characteristics in EAE. As described by Becher et al., CD40 expressed by CNS-endogenous cells controls the migration and retention of MOG-reactive T cells in the CNS of mice during EAE [20]. It is possible that our SMI does not cross the BBB and only affects peripheral monocytes and not the microglia, which in turn could explain why we only see a reduction in infiltrating macrophages and not in T cells. Therefore, it could be interesting to investigate whether cell type-specific delivery of our SMI to microglia may be an attractive strategy to increase its efficacy in vivo. A nanomedicine-based approach could be of interest to achieve delivery of the inhibitors across the BBB to microglia [53].

## Conclusions

In conclusion, we have shown that small molecule-mediated inhibition of the CD40-TRAF6 interaction limited ROS production by human monocytes and reduced migration of human monocytes across an in vitro BBB. The CD40-TRAF6 SMI reduced severity of symptoms of EAE in rats, but not in mice, suggesting that inhibiting monocyte-derived macrophage infiltration into the CNS is not sufficient to fully prevent clinical symptoms of EAE. Our SMI can therefore be considered as co-treatment to inhibit monocyte recruitment in MS.

## Additional files

Additional file 1: Figure S1. Immune cell accumulation in the spinal cords of EAE rats is reduced by SMI 6877002 treatment. Gene expression in the rat spinal cord after recovery was measured by qPCR. mRNA expression levels of *TNF*, *NOS2*, *MMP2*, *MMP9*, and *CD80* presented as relative expression compared to *GAPDH*. Experiments were performed in eight animals per group, after recovery of EAE. Results are presented as the mean ± SEM, **P < 0.01 as determined by Student’s *t* test. (TIF 15382 kb)

Additional file 2: Figure S2. Flow cytometry of circulating leukocytes is not affected by SMI treatment. Immune cell subsets measured in (A) blood and (B) spleen. Experiments were performed with six animals of either EAE or SMI group and three animals in the control group. Results are presented as the mean ± SEM. (TIF 78393 kb)

Additional file 3: Figure S3. mRNA gene expression at the peak of EAE in the spinal cord of mice is not affected by SMI 6877002 treatment. *TNF*, *IFN-γ*, *MCP-1*, *IL-17*, *IL-10*, and *FoxP3* mRNA gene expression in the spinal cord determined by real-time quantitative PCR and presented as relative expression compared to *GAPDH*/*CycloA*/*Rplp0*. Experiments were performed with six animals of either EAE or SMI group and three animals in the control group. Results are presented as the mean ± SEM,*P < 0.05, **P < 0.01, ***P < 0.001, as determined by Student’s *t* test. (TIF 23399 kb)

## Abbreviations

BBB: Blood-brain barrier
BMVECs: Brain microvascular endothelial cells
BSA: Bovine serum albumin
CD: Cluster of differentiation
CFA: Complete Freud’s adjuvant
CNS: Central nervous system
DHR: Dihydrorhodamine
EAE: Experimental autoimmune encephalomyelitis
ECM: Extracellular matrix
EGF: Endothelial growth factor
FBS: Fetal bovine serum
FGF: Fibroblast growth factor
IGF: Insulin-like growth factor
MBP: Myelin basic protein
MMP: Matrix metalloproteinase
MS: Multiple sclerosis
PBS: Phosphate-buffered saline
ROS: Reactive oxygen species
SMI: Small molecule inhibitor
TRAF: TNF receptor-associated factor
VEGF: Vascular endothelial growth factor
VLA: Very late antigen
