# Supplementary figures and images for: Inhibition of CD40-TRAF6 interactions by the small molecule inhibitor 6877002 reduces neuroinflammation
Source: J Neuroinflammation. 2017 May 12;14:105. doi: 10.1186/s12974-017-0875-9 (PMC5427621; doi:10.1186/s12974-017-0875-9)

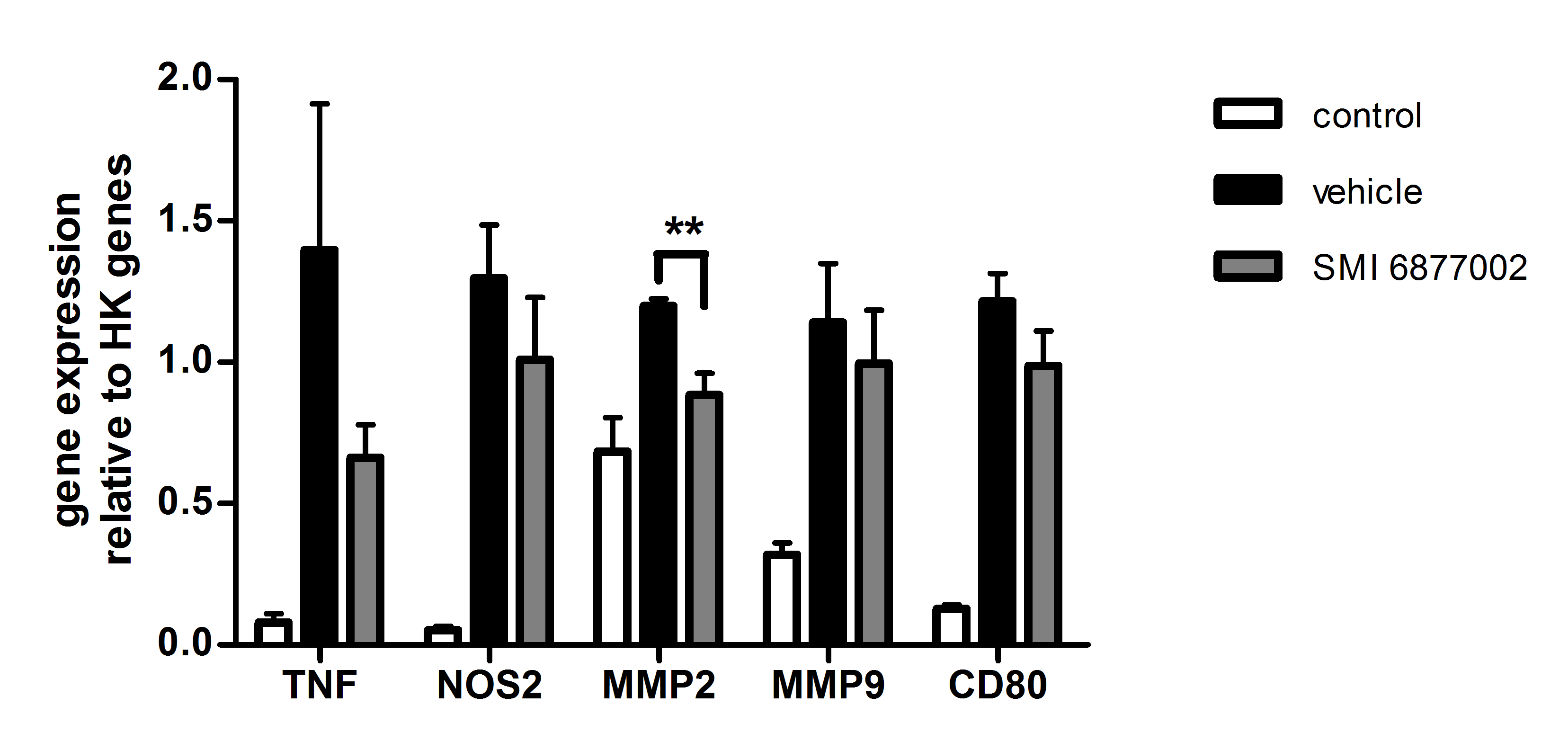

Supplement: Supplementary file 1 — Immune cell accumulation in the spinal cords of EAE rats is reduced by SMI 6877002 treatment. Gene expression in the rat spinal cord after recovery was measured by qPCR. mRNA expression levels of TNF, NOS2, MMP2, MMP9, and CD80 presented as relative expression compared to GAPDH. Experiments were performed in eight animals per group, after recovery of EAE. Results are presented as the mean ± SEM, **P < 0.01 as determined by Student’s t test. (TIF 15382 kb) [file 12974_2017_875_MOESM1_ESM.tif]

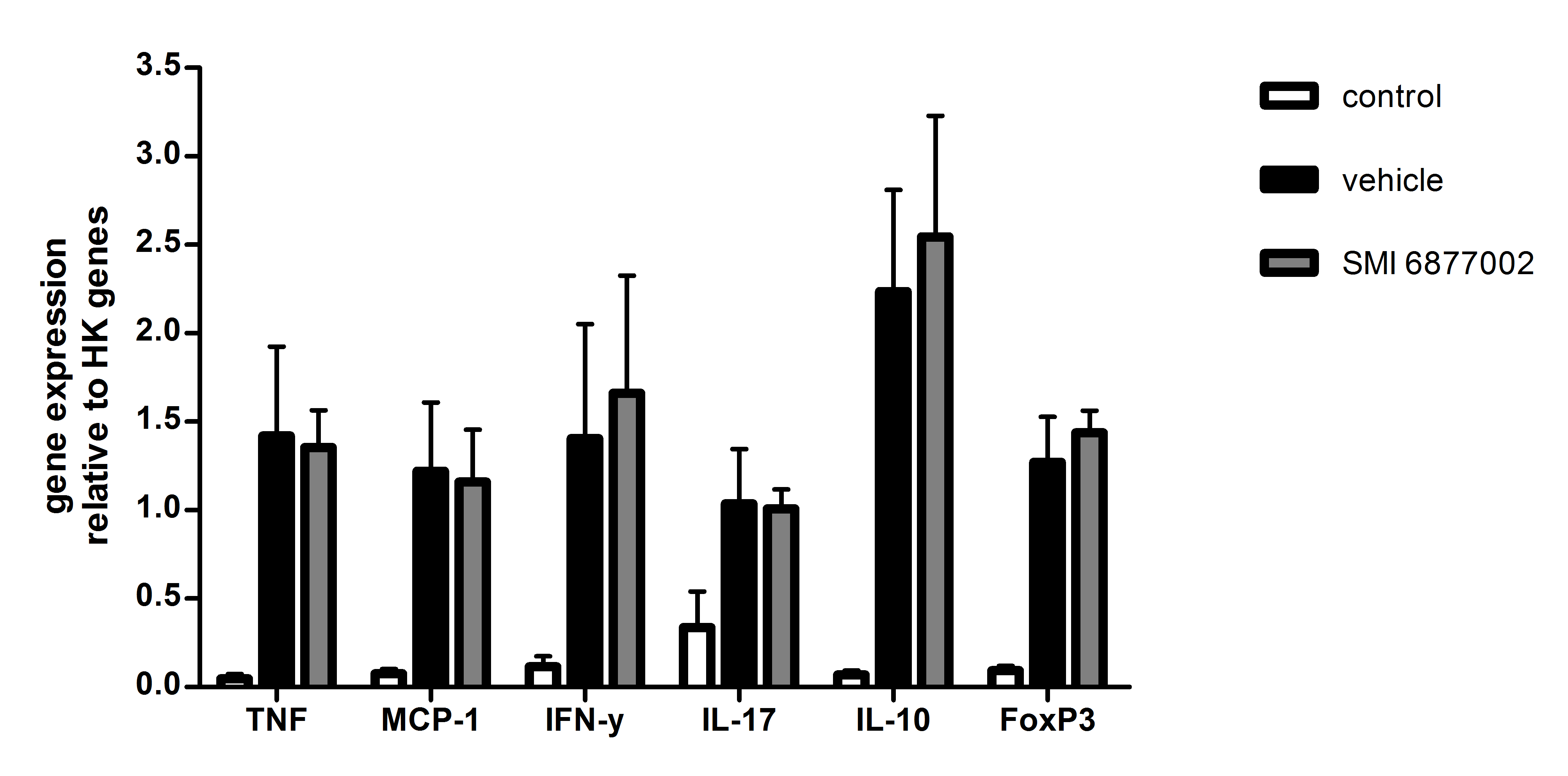

Supplement: Supplementary file 3 — mRNA gene expression at the peak of EAE in the spinal cord of mice is not affected by SMI 6877002 treatment. TNF, IFN-γ, MCP-1, IL-17, IL-10, and FoxP3 mRNA gene expression in the spinal cord determined by real-time quantitative PCR and presented as relative expression compared to GAPDH/CycloA/Rplp0. Experiments were performed with six animals of either EAE or SMI group and three animals in the control group. Results are presented as the mean ± SEM,*P < 0.05, **P < 0.01, ***P < 0.001, as determined by Student’s t test. (TIF 23399 kb) [file 12974_2017_875_MOESM3_ESM.tif]
